# The Effect of Gestational and Lactational Age on the Human Milk Metabolome

**DOI:** 10.3390/nu8050304

**Published:** 2016-05-19

**Authors:** Ulrik K. Sundekilde, Eimear Downey, James A. O’Mahony, Carol-Anne O’Shea, C. Anthony Ryan, Alan L. Kelly, Hanne C. Bertram

**Affiliations:** 1 Department of Food Science, Aarhus University, Årslev 5792, Denmark; hannec.bertram@food.au.dk; 2 School of Food and Nutritional Sciences, University College Cork, Cork T12 YN60, Ireland; eimeardowney@gmail.com (E.D.); sa.omahony@ucc.ie (J.A.M.); a.kelly@ucc.ie (A.L.K.); 3 Department of Paediatrics and Child Health, University College Cork, Cork T12 YN60, Ireland; Ca.OShea@ucc.ie (C.A.S.); tony.ryan@ucc.ie (C.A.R.)

**Keywords:** pre-term, infant, nutrition, human milk, metabolites, NMR, metabolomics

## Abstract

Human milk is the ideal nutrition source for healthy infants during the first six months of life and a detailed characterisation of the composition of milk from mothers that deliver prematurely (<37 weeks gestation), and of how human milk changes during lactation, would benefit our understanding of the nutritional requirements of premature infants. Individual milk samples from mothers delivering prematurely and at term were collected. The human milk metabolome, established by nuclear magnetic resonance (NMR) spectroscopy, was influenced by gestational and lactation age. Metabolite profiling identified that levels of valine, leucine, betaine, and creatinine were increased in colostrum from term mothers compared with mature milk, while those of glutamate, caprylate, and caprate were increased in mature term milk compared with colostrum. Levels of oligosaccharides, citrate, and creatinine were increased in pre-term colostrum, while those of caprylate, caprate, valine, leucine, glutamate, and pantothenate increased with time postpartum. There were differences between pre-term and full-term milk in the levels of carnitine, caprylate, caprate, pantothenate, urea, lactose, oligosaccharides, citrate, phosphocholine, choline, and formate. These findings suggest that the metabolome of pre-term milk changes within 5–7 weeks postpartum to resemble that of term milk, independent of time of gestation at pre-mature delivery.

## 1. Introduction

Human milk (HM) is the recognised gold standard for feeding new-born full-term healthy infants. HM is a unique food source that contains all the exacting amounts of required nutrients to support the growth and development of term infants during the first six months of life. The health benefits of HM have been well documented [1]. Current data suggest that the quantity of HM consumed by healthy term infants is on average 0.778 kg human milk/day, with males consuming 0.056 kg/day more than females. Human milk consumption rises rapidly during the first month postpartum to ~0.6 kg/day and increases to ~0.82 kg/day in 3–4 month old infants [2].

Human milk is also regarded as particularly important for feeding pre-mature infants. There are many health benefits related to providing a pre-term infant with HM, including improvements in digestion, nutrient absorption, gastrointestinal function, and neurodevelopment of the infant [3,4]. In addition, an exclusive HM diet has been associated with lower rates of necrotizing enterocolitis, a potentially fatal gastrointestinal complication, in premature infants [5]. Human milk oligosaccharides (HMO) are known to affect the gut microbiota and it has been speculated that HMOs could account for the lower observed incidences of necrotizing enterocolitis [6].

Mother’s milk is inadequate for rapidly growing pre-term neonates, which can be ascribed to a higher protein and energy requirement of pre-term neonates, particularly those of low birth weight and <28 weeks gestation, leading to insufficient weight gain and nutrition deficits [7,8]. For extremely pre-term and very low birth weight infants, the fortification of HM using commercial fortifiers is often recommended in order to supply the neonate with the nutrients required to support its rapid rate of growth. Studies have shown that the addition of HM fortifier can improve weight, length, and head circumference growth, in addition to bone mineralization and neurological outcomes [9,10,11,12,13]. 

It is known that the composition of pre-term milk differs from that of milk of full-term mothers, with higher reported levels of total protein, fat, carbohydrate and energy in pre-term milk [14]. Differences in the proteome of the two types of milk have also been reported [15]. Generally, studies have focused on the macronutrient content of pre-term and term HM without consideration of micronutrients such as biologically significant metabolites [14].

Previous work on milk metabolomes has predominantly considered bovine milk [16,17,18,19,20] and human milk [21,22,23,24,25,26,27,28]. Recently, a multi-analytical platform study identified 710 metabolites in human milk by using a combination of MS- and NMR-based analytical techniques [21]. Moreover, a recent study identified subtle differences between some milk metabolites during the first month of lactation [27]. However, the changes in pre-term and full-term human milk metabolomes over a full lactation time course have not yet been reported in detail.

In this study, we report how gestational age affects the low-molecular-weight metabolome of HM from mothers of pre-term and term infants over range of stages of lactation. The milk metabolomes were compared using proton nuclear magnetic resonance spectroscopy (^1^H NMR)-based metabolomics from milk samples from mothers at different gestational ages. Longitudinal samples were also examined to analyse how pre-term milk develops with time postpartum compared with term milk.

## 2. Materials and Methods 

### 2.1. Ethical Approval

Ethical approval for this study was granted by The Clinical Research Ethics Committee of the Cork Teaching Hospitals, Cork, Ireland (clinical number reference ECM 4(s) 06/08/13).

### 2.2. Samples and Sample Collection

#### 2.2.1. Pre-term Samples

Multiple frozen (−20 °C) longitudinal pre-term HM samples were collected from 15 individual mothers of pre-term infants (Table 1). The HM samples were collected from the freezers of the neonatal intensive care unit of Cork University Maternity Hospital (Wilton, Co. Cork, Ireland). Each bottle of HM was dated allowing for an accurate calculation of the gestational age of the infant when the milk was expressed. The HM samples from each individual mother were pooled according to ‘day postpartum’ up to 14 days and according to ‘week postpartum’ after this, creating *n* = 62 pre-term HM samples. For one donor (Pre-1), information on number of days postpartum was unavailable and accordingly samples from this donor were removed from multivariate models using gestational age or days postpartum for modelling or visualization purposes.

#### 2.2.2. Full-term Samples

Frozen (−20 °C) HM samples (*n* = 30) were obtained from The Western Trust Milk Bank, Irvinestown, Co. Fermanagh, Ireland (Table 2). The HM samples were from 30 individual mothers of healthy full-term infants who donated milk with consent for use for research purposes. The mothers expressed milk in a domestic setting and stored milk at −20 °C before shipping to The Western Trust Milk Bank, Ireland. HM was pasteurised and microbiologically screened before being frozen and shipped to University College Cork where it was stored at −20 °C. Milk samples were categorized according to the length of time postpartum; colostrum (<5 days postpartum; *n* = 5), transitional (6 days–2 weeks postpartum; *n* = 4), mature (>2 weeks, *n* = 21).

### 2.3. NMR Spectroscopy

NMR spectroscopy was essentially performed as described earlier [29]. Briefly, the samples skimmed by centrifugation at 4000 *g* for 15 min and removal of the top fat layer before filtering to remove residual lipids and protein using Amicon Ultra 0.5 mL 10 kDa (Millipore, Billerica, MA, USA) spin filters at 10,000 *g* for 30 min at 4 °C. A filtered sample (500 µL) was mixed with 100 µL D_2_O containing 0.025% 3-(trimethylsilyl) propionic-2,2,3,3-d_4_ acid, sodium salt (TSP; Sigma-Aldrich, St. Louis, MO, USA) as an internal chemical shift reference. ^1^H NMR spectroscopy was performed at 298 K on a Bruker Avance III 600 spectrometer, operating at a ^1^H frequency of 600.13 MHz, and equipped with a 5-mm ^1^H TXI probe (Bruker BioSpin, Rheinstetten, Germany). The sample sequence was randomized prior to acquisition and standard one-dimensional spectra were acquired using a single 90° pulse experiment with a relaxation delay of 5 s. Water suppression was achieved by irradiating the water peak during the relaxation delay, and a total of 64 scans were collected into 32,768 data points spanning a spectral width of 12.15 ppm. All ^1^H spectra were initially referenced to the TSP signal at 0 ppm. Prior to Fourier transformation, the data were multiplied by a 0.3 Hz line-broadening function. The proton NMR spectra were phase and baseline corrected manually using Topspin 3.2 (Bruker Biospin, Rheinstetten, Germany). NMR signals were assigned in accordance with existing literature [20,21,25,29], 2D NMR spectroscopy, Chenomx NMR Suite 8.1.2 (Chenomx Inc, Edmonton, AB, Canada) and the Human Metabolome Database [30]. 

### 2.4. Secretor Status

Maternal secretor status was determined as previously described [25]. In brief the absence or presence of 2-FL was used to determine secretor status quantified by NMR spectroscopy (2-FL corresponds to NMR signal at δ 5.32 ppm). Milk from mothers classified as non-secretors did not have any detectable levels of 2-FL, whereas milk from mothers classified as secretors did contain 2-FL.

### 2.5. Multivariate Data and Statistical Analyses

NMR spectra of milk samples were aligned using Icoshift by co-shifting of the whole spectra according to the anomeric lactose proton at 5.23 ppm [31]. The proton NMR spectra were subdivided into 0.01 ppm bins, reducing each spectrum into 957 separate variables in the regions 10.00–5.00 and 4.72–0.5 ppm. Principal component analysis (PCA) and orthogonal partial least squares discriminant analysis (OPLS-DA) were performed in order to identify differences in the metabolite profiles. The data was mean-centred and Pareto-scaled prior to analysis. The OPLS-DA model was cross-validated using segmentation with seven splits. Covariance was investigated by analysis of OPLS-DA regression coefficients back-transformed to original data and colour coded by the loading weights [32]. The multivariate data analysis was performed using SIMCA-P + 13 (Umetrics AB, Umeå, Sweden). Alignment by Icoshift, binning, and analysis of OPLS-DA plots were performed in MATLAB 7.13 using in-house developed scripts (MathWorks Inc., Natick, MA, USA). Univariate statistical significance was evaluated by Student’s *t*-test using the Statistics Toolbox in MATLAB 7.13 (MathWorks Inc., Natick, MA, USA). 

## 3. Results

HM samples (*n* = 92) were collected from 45 individual mothers with different gestational ages at delivery, ranging from 24.6–35.8 weeks in the case of pre-term samples and more than 37 weeks for full-term samples (Table 1 and Table 2). For pre-mature deliveries, infant birth weight ranged from 540 g to 3260 g. Age of the mothers of pre-term infants ranged from 26–45 years, and all had at least one previous pregnancy longer than 24 weeks gestation (Table 1). 

**Figure 1:**
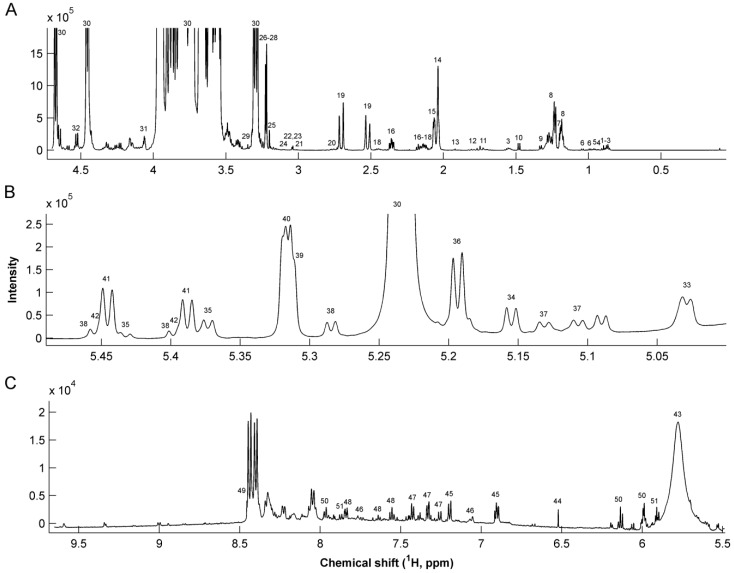
Median ^1^H NMR spectrum of 92 human milk samples. (**A**) Aliphatic region 4.6–0 ppm (**B**) Human milk oligosaccharides in 5.45–5.00 ppm region; and (**C**) aromatic region 9.7–5.5 ppm region. For peak assignments refer to Table 3.

The median spectrum of all 92 milk samples is shown in Figure 1. Each signal corresponds to proton resonances in milk metabolites according to assignments in Table 3. Differences in fucosylated human milk oligosaccharides (HMO) have previously been described to depend on maternal secretor status [33]. Accordingly, secretor status of the mothers was determined by examining the specific patterns of fucosylated oligosaccharides (Figure 1B) [25]. Thirteen mothers were found to be non-secretors, accounting for 28.9% of the mothers included in the study (Table 1 and Table 2).

**Figure 2:**
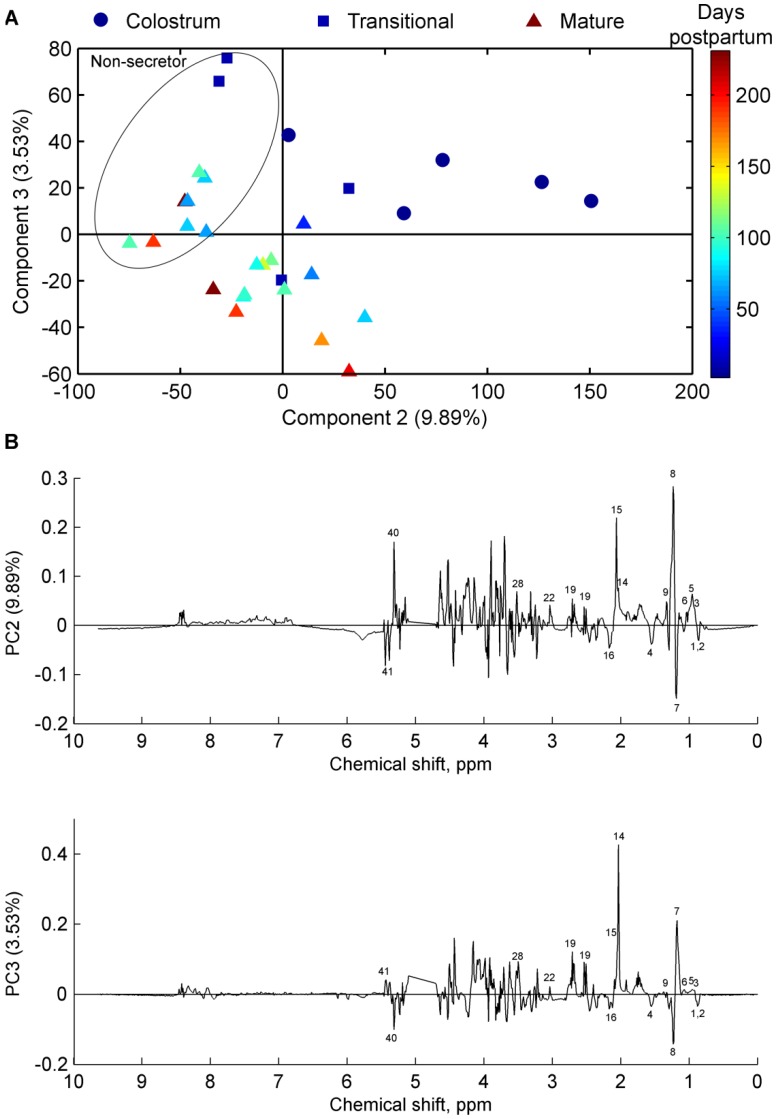
(**A**) Principal component analysis scores plot of full-term milk samples from 30 mothers (*n* = 30)); samples are coloured according to days postpartum; Colostrum (dots), transitional (squares), and mature (triangles); the circle denotes samples from non-secretor mothers, while remaining samples are from secretor mothers; and (**B**) corresponding loading line plots. For peak assignments refer to Table 3.

**Figure 3:**
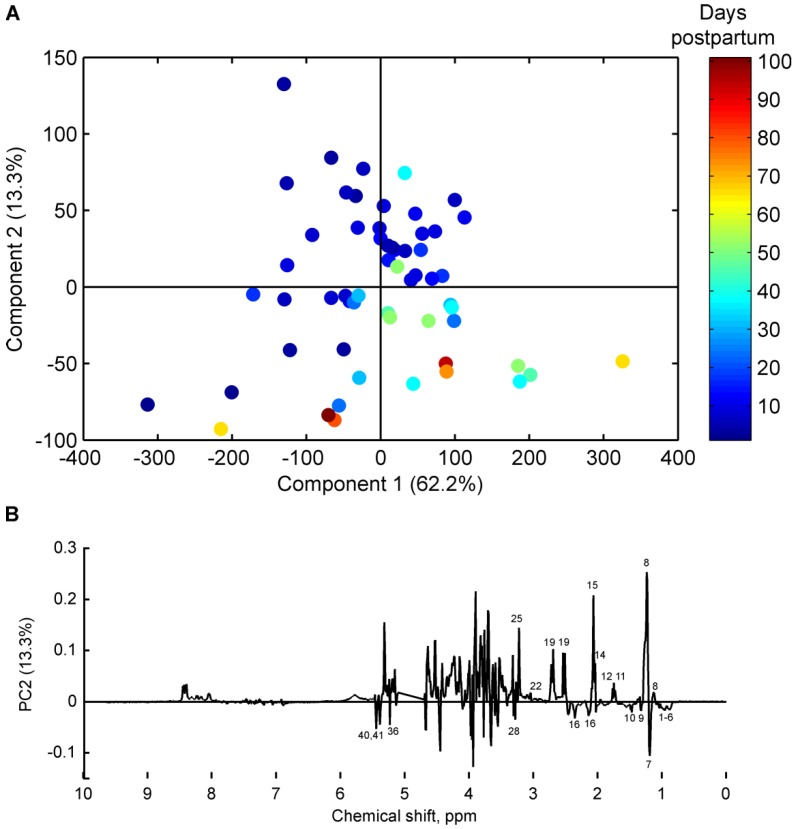
(**A**) Principal component analysis scores plot of pre-term milk samples from 15 mothers (*n* = 58), coloured according to number of days postpartum; and (**B**) corresponding loading line plot. For peak assignments refer to Table 3.

The collected milk samples were analysed for changes during lactation following both full-term (Figure 2) and pre-term (Figure 3) delivery. Figure 2 shows a scores plot of principal component (PC) 2 and PC3 from a principal component analysis (PCA) model of full-term milk samples. The samples are grouped into colostrum, transitional, and mature groups and coloured according to days postpartum. The scores plot shows the distinct groupings of the milk samples, and indicates a gradual change in the milk metabolome as the milk develops from colostrum towards mature milk (Figure 2). In addition, no detectable differences were observed between mature milk samples in the intervals before 26 weeks and after 26 weeks (Figure 2). There was an apparent difference in milk metabolites depending on secretor status in term milk samples, as the milk samples were separated into secretor or non-secretor groups along PC2 (Figure 2).

Several milk metabolites were found to be present in significantly different concentration in colostrum, transitional, and mature milk. Fucosylated oligosaccharides, and also components of oligosaccharides (Fucose, *N*-acetylneuraminic acid, *N*-acetylglucosamine), were found at the highest levels in colostrum, and levels decreased in mature milk samples (Figure 2B). Moreover, levels of valine, leucine, pantothenate, citric acid, lactic acid, betaine, and creatinine were higher in colostrum and transitional milk compared with mature HM, and levels of glutamate, butyrate, caprylate, and caprate were higher in mature HM compared with colostrum and transitional milk. The level of β-hydroxybutyrate was found to be independent of milk maturity in full-term HM (Figure 2B). Milk from non-secretor mothers did not contain oligosaccharides with α1-2 fucosylated structures (Figure 2B); however, the level of 3’ fucosyllactose (3’-FL) was increased in non-secretor mothers compared with secretor mothers. 

Similarly, the pre-term milk samples were also examined for an effect of stage of lactation on the milk metabolome. The scores plot of a two component PCA model of milk samples from pre-term mothers is shown in Figure 3, in which samples are coloured according to number of days postpartum. The scores and loadings plots clearly show the changes in milk metabolome occurring over time in pre-term milk. Levels of fucosyl moieties, *N*-acetylneuraminic acid, *N*-acetylglucosamine, 3’-sialyllactose, 6’-sialyllactose, 2’-fucosyllactose, citric acid, choline, and creatinine decreased with time postpartum (Figure 3B), while levels of 3-FL, lacto-*N*-difucohexaose I (LNDFH I), butyrate, caprylate, caprate, lactic acid, valine, leucine, alanine, glutamate, and pantothenate increased with time postpartum (Figure 3B). Only two mothers, who donated seven milk samples in total, were identified as non-secretors (Table 1). Thus, it was not possible to identify differences in the PCA model for pre-term samples on the basis of secretor status.

**Figure 4:**
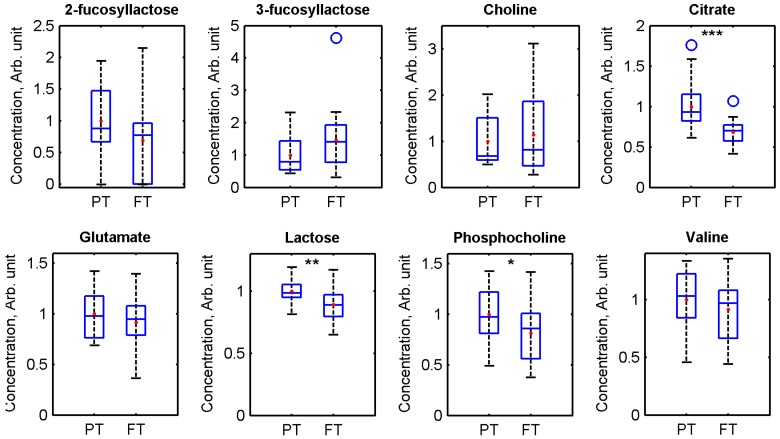
Box plots showing the distributions of 2-fucosyllactose, 3-fucosyllactose, choline, citrate, glutamate, lactose, phosphocholine, and valine concentrations in pre-term (PT) and full-term (FT) milk. Pre-term milk samples <14 days postpartum and full-term colostrum, transitional, and mature >26 weeks were excluded from the analysis in order to compare milk with a similar range of days postpartum. Horizontal lines indicate medians; coloured boxes specify interquartile ranges and dashed lines the ranges without outliers. The open circles indicate outliers (falls in-between 1.5× and 3× the interquartile range). *** = *P* < 0.001, ** = *P* < 0.01, * = *P* < 0.05; the comparisons were made using two-tailed Student’s *t*-test.

As some of the same time-related changes were apparent in both pre-term and full-term milk samples, the difference between the pre-term and full-term milk metabolomes of similar days postpartum was investigated. Citrate (*P* = 0.00057), lactose (*P* = 0.0039), and phosphocholine (*P* = 0.049 were found to be present in significantly higher levels in pre-term milk samples compared with full-term milk samples (Figure 4). Citrate level is known to decrease with time postpartum [28], while lactose is the major milk metabolite identified by NMR spectroscopy [20]. 

**Figure 5:**
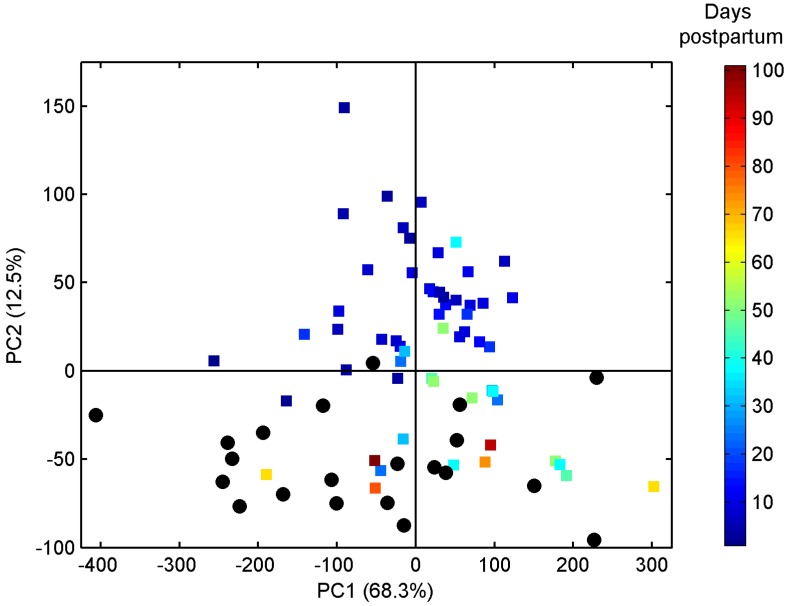
Principal component analysis scores plot of mature milk samples from pre-term (squares, *n* = 58) and full-term (circles, *n* = 30) mothers. Pre-term milk samples are coloured according to days postpartum.

Thus, in the following analysis, NMR resonances originating from lactose were removed in order to increase the weight of other metabolites in the multivariate models. A PCA of pre-term and full-term milk samples (*n* = 88) was generated using two principal components which, together, explained 80.8% of the total variance described by the PCA model (Figure 5). From the PCA model, it is apparent that the milk metabolite profile from mothers of pre-term infants older than 5–7 weeks resembles that of full-term milk, as they are positioned in close proximity to the full-term milk samples, independent of time of gestation at delivery (Figure 5). 

**Figure 6:**
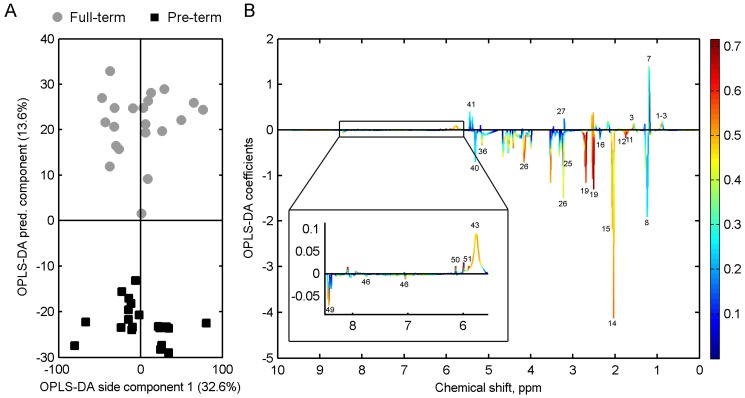
(**A**) Orthogonal partial least squares discriminant analysis of pre-term (*n* = 20, days postpartum range 3–14 weeks) and full-term (*n* = 21, days postpartum range 3–26 weeks) milk. Full-term colostrum, transitional, and pre-term milk <2 weeks postpartum have been excluded. Cross-validation, Q2: 0.70; (**B**) corresponding OPLS-DA coefficients plot. Each variable has been coloured according to the OPLS-DA loadings (correlation between NMR variables and pre-term/full-term classes); for peak assignments refer to Table 3.

OPLS-DA was also performed on the pre-term and full-term milk samples (Figure 6). Full-term and pre-term colostrum and transitional milk were excluded from the analysis, due to there being too few samples of these, and to be able to compare milk samples with similar days postpartum. The OPLS-DA model shows that significant differences exist between the metabolite profiles of pre-term and full-term milk (Figure 6B). In full-term milk, carnitine, caprylate, caprate, pantothenate, beta-hydroxybutyrate, and urea were found to be present in higher levels compared with pre-term milk, while lactose (Figure 4), Fucosyl moieties, *N*-acetylneuraminic acid, *N*-acetylglucosamine, 3’-sialyllactose, 6’-sialyllactose, lacto-*N*-difucohexaose I (LNDFH I), glutamate, citric acid, phosphocholine, choline, and formic acid were found in higher levels in pre-term milk (Figure 6).

## 4. Discussion

Pre-mature birth is considered to have long-lasting adverse effects on health, and it has been proposed that the introduction of metabolomics technologies may facilitate better understanding of these effects [34]. The exact causes of preterm delivery are not clear, but often associated with inflammation [35]. In the present study, causes of the preterm delivery were not known. In this study, differences in metabolomic profiles of HM were associated with gestational age. Previous studies have shown many health benefits of breast-feeding pre-term infants. However, mother’s milk may not always be adequate for pre-term infants with high nutrition density requirements, which can lead to insufficient weight gain and nutrition deficits. To date, only a few metabolomics studies have been performed on pre-term human milk in the first few weeks of lactation [27]. To our knowledge, this is the first study to comprehensively characterize and compare the pre-term and full-term human milk metabolome over a lactation time-course (up to 14 weeks postpartum). Milk metabolomics may be used in a clinical setting [23], in order to establish the milk metabolome for infant nutrition in cases where the optimal nutrition is a key advantage as in pre-term infants. Milk metabolomics studies in cows’ milk have previously shown an ability to yield information about the health status of the cow [29,36]. It can likewise be hypothesized that the milk metabolome of lactating mothers can give information that can be used in a clinical setting.

Previous studies have shown that several factors influence the variability in the milk metabolome. The epithelial cells in the mammary gland are accountable for the milk production, and these cells are ultimately responsible for converting most precursors into milk components, but many other cell types are also involved in milk production [37]. Development of the mammary epithelial cells during pregnancy has been shown to influence transport pathways in the mammary gland responsible for different protein profiles of milk [38]. Moreover, insulin has been shown to be actively transported into milk and maternal diabetes affect milk insulin levels [39]. Maternal factors such as diet, lifestyle and phenotype have been shown to influence the milk composition [26]. Moreover, secretor status is also important for the biosynthesis of milk oligosaccharides [26]. The presence of α1,2 fucosyltransferase gene (FUT2) is important for the milk oligosaccharides since non-secretors, which lack the FUT2 gene, are unable to biosynthesize HMOs with a fucose bound to a *N*-acetylglucosamine via a glycosidic α1,2 linkage. Non-secretor status occurs in approximately 20% of most human populations [40,41]. In the present study, non-secretor status, identified by increased level of 3-FL, was found in 28.9 % of milk donors. Gestational age and the stage of lactation are also known to influence the milk proteins [14], lipids [42], and lactose [43]. Preliminary HM metabolomic studies have shown a difference between the metabolite profiles of pre-term and full-term HM [22,24]. Additionally, the metabolite extraction procedure in sample preparation prior to NMR spectroscopy has shown to influence the milk metabolite levels. In a recent paper, a sample preparation method using ultrafiltration as employed in the present study, was found to be superior compared with methanol/chloroform extraction in terms of separating small molecules from proteins and lipids [28]. Levels of HMO and its components are known to decrease with time [44]. A specific HMO, lacto-*N*-tetraose, has also been shown to be elevated in pre-term milk [45]. Interestingly, specific HMOs, LNDFH I, 3’-sialyllactose, and 6’-sialyllactose, and common components of HMO, including fucose, *N*-acetylglucosamine, and *N*-acetylneuraminic acid were identified to be present in higher concentrations in pre-term milk. HMOs have previously been shown to prevent necrotizing enterocolitis in neonatal rats [6], which may be linked to alterations in the microbiota of the infant’s gut [46]. Thus, the elevated amounts of HMOs in pre-term milk may be a factor in the observed association of lower rates of necrotizing enterocolitis in premature infants fed HM [5].

Most of the free amino acids in HM steadily decrease in level as the milk matures [42]. However, the pre-term milk amino acid composition has not been well characterized throughout the course of lactation [47,48,49]. Glutamate is known to increase with lactation time [47] and this was also found in both pre-term and full-term milk in the present study. Valine and leucine, two essential amino acids, were found at the highest concentration in full-term colostrum and decreased with time postpartum, in agreement with other human milk metabolomics studies [27]. In contrast, increases in valine and leucine levels were observed in pre-term milk with time post-partum, which is in contrast to the pattern observed in full-term milk and that previously reported for pre-term milk [48,49].

Short- and medium-chain fatty acids (SMCFA; butyrate, caprylate, and caprate) were found in higher concentrations in full-term milk compared with pre-term milk. Moreover, the levels of SMCFA increased with time postpartum, as previously reported [50]. Choline is of high nutritional value, as it is abundant in cell membranes and acts as a precursor for the important messenger acetylcholine, and infants require high amounts of choline in order to sustain their rapid growth. Phosphocholine was found in higher levels in pre-term milk than in full-term milk, which is in agreement with previous reports on phosphocholine [51]. In contrast, pre-term milk has previously been shown to contain lesser amounts of choline [51], which is contradictory to findings in the present study.

In this study, it was shown that, after 5–7 weeks the metabolite profiles of pre-term milk resembled that of full-term milk, independently of gestational age. For extremely early pre-term infants born at a gestational age of, e.g., 24 weeks, these findings suggest that mother’s milk becomes equivalent to mature milk for term infants by the time the infant reaches ~29 weeks post-menstrual age. This is a significant finding concerning a period in which the infant still requires advanced nutritional support to maintain its growth and long-term developmental requirements. Clinical metabolomics could potentially be advantageous to determine when milk metabolites levels become inadequate due to maturation of milk, if causal relationships between milk metabolites and infant growth parameters can be established. Previous studies have shown that protein concentration remains at higher levels in pre-term human milk even after 8 weeks of lactation [13]; however, we believe this to be the first study to document the changes in the metabolomics profile of pre-term human milk as it evolves to resemble that of full-term milk, and to show that this occurs independent of gestational age at delivery.

In conclusion, specific differences in milk metabolites exist between milk from mothers delivering pre-maturely and at term. Moreover, milk metabolite composition is associated with gestational age and the metabolome of pre-term milk changes within 5–7 weeks postpartum to resemble that of full-term milk, independent of time of gestation at delivery. However, as this study did not systematically collect samples from a cohort of mothers in a longitudinal design, further studies of this kind are recommended to more fully understand the significance of the changes reported herein.

## Figures and Tables

**Table 1:** Sample information from mothers of pre-term infants.

Sample Id	Secretor Status	Gestation	Infant Weight, Grams	Age of Mother, Years	Gravida ^1^	Para ^2^	Number of Samples	Postpartum Span ^3^
Pre 01	Se^+^	–	–	–	–	–	4	–
Pre 02	Se^+^	38 weeks	3210	35	2	2	4	7–11 weeks
Pre 03	Se^+^	28 weeks	1190	33	1	1	9	4–12 days
Pre 04	Se^+^	31 weeks	1830	36	3	3	5	1–5 weeks
Pre 05	Se^+^	30 weeks	1340	29	1	1	7	4 days–7 weeks
Pre 06	Se^+^	32 weeks + 2 days	2340	42	2	2	1	8 days
Pre 07	Se^−^	24 weeks + 4 days	540	33	1	1	4	1–4 days
Pre 08	Se^−^	31 weeks	1680	35	2	2	2	5–14 weeks
Pre 09	Se^+^	35 weeks + 6 days	2410	33	1	1	3	2–5 weeks
Pre 10	Se^+^	35 weeks + 2 days	3100	26	1	1	2	4–5 days
Pre 11	Se^+^	33 weeks + 4 days	1800	29	6	5	2	4–6 days
Pre 12	Se^+^	35 weeks + 4 days	3260	45	4	3 + 2 ^4^	2	9–10 days
Pre 13	Se^+^	26 weeks	650	40	–	–	15	6 days–8 weeks
Pre 14	Se^−^	28 weeks + 3 days	–	–	–	–	1	13 weeks
Pre 15	Se^+^	32 weeks	–	–	–	–	1	9 weeks

^1^ Number of pregnancies, including the current pregnancy; ^2^ number of times the mother has given birth; ^3^ time range indicates days postpartum of first and last samples from that donor; ^4^ indicates twin pregnancy.

**Table 2:** Sample information from mothers of term infants.

Sample ID	Secretor status	Time postpartum	Group
Term 01	Se^+^	<5 days	Colostrum
Term 02	Se^+^	<5 days	Colostrum
Term 03	Se^+^	<5 days	Colostrum
Term 04	Se^+^	<5 days	Colostrum
Term 05	Se^+^	<5 days	Colostrum
Term 06	Se^+^	2 weeks	Transitional
Term 07	Se^+^	2 weeks	Transitional
Term 08	Se^−^	6 days	Transitional
Term 09	Se^−^	2 weeks	Transitional
Term 10	Se^−^	15 weeks	Mature
Term 11	Se^−^	9 weeks	Mature
Term 12	Se^+^	15 weeks	Mature
Term 13	Se^−^	11 weeks	Mature
Term 14	Se^−^	15 weeks	Mature
Term 15	Se^−^	11 weeks	Mature
Term 16	Se^+^	16 weeks	Mature
Term 17	Se^−^	9 weeks	Mature
Term 18	Se^+^	19 weeks	Mature
Term 19	Se^+^	24 weeks	Mature
Term 20	Se^+^	11 weeks	Mature
Term 21	Se^+^	7–10 weeks	Mature
Term 22	Se^+^	13 weeks	Mature
Term 23	Se^+^	13 weeks	Mature
Term 24	Se^+^	14 weeks	Mature
Term 25	Se^+^	5 weeks	Mature
Term 26	Se^+^	27 weeks	Mature
Term 27	Se^−^	33 weeks	Mature
Term 28	Se^−^	27 weeks	Mature
Term 29	Se^+^	29 weeks	Mature
Term 30	Se^+^	33 weeks	Mature

**Table 3:** List of metabolites in human milk with chemical shifts in ppm from internal TSP standard and assignment of resonances.

#	Metabolite	^1^H Chemical shift (ppm)	Assignment	#	Metabolite	^1^H Chemical shift (ppm)	Assignment
1	Caprylate	0.85	CH_3_	29	Methanol	3.37	CH_3_
		1.53	CH_2_	30	Lactose	3.23, 3.5–4.0	Multiple
2	Caprate	0.85	CH_3_	31	Gluconate	4.05	CH
		1.53	CH_2_	32	Galactose	4.57	CH
3	Butyrate	0.88	CH_3_	33	Fuc α1,4 GlcNAc	5.03	CH-1
		1.55	β-CH_2_	34	Fuc α1,3 GlcNAc	5.19	CH-1
		2.16	α-CH_2_	35	LNDFH II	5.03	Fuc (α1-4) CH-1
4	Pantothenate	0.91	CH_3_			5.38	Fuc (α1-3) αGlc CH-1
5	Leucine	0.94	CH_3_			5.43	Fuc (α1-3) βGlc CH-1
6	Valine	0.98	γ-CH_3_	36	LNDFH I	5.16	Fuc (α1-2) CH-1
		1.03	γ’-CH_3_			5.03	Fuc (α1-4) CH-1
7	3-BHBA	1.18	CH_3_	37	LNFP III	5.11	Fuc (α1-3) GlcNAc CH-1
		2.39	CH_2_			5.13	Fuc (α1-3) GlcNAc CH-1
8	Fucosyl moieties	1.19	CH_3_-6	38	LDFT	5.29	Fuc (α1-2) CH-1
		1.23–1.29	CH_3_-6			5.4	Fuc (α1-3) αGlc CH-1
9	Lactate	1.32	CH_3_			5.46	Fuc (α1-3) βGlc CH-1
10	Alanine	1.47	CH_3_	39	LNFP I	5.32	Fuc (α1-2) CH-1
11	6’-SL	1.73	CH_3_	40	2’FL	5.32	Fuc (α1-2) CH-1
12	3’-SL	1.78	CH_3_	41	3’FL	5.39	Fuc (α1-3) αGlc CH-1
13	Acetate	1.91	CH_3_			5.44	Fuc (α1-3) βGlc CH-1
14	GlcNAc	2.04	CH_3_	42	LNFP V	5.39	Fuc(α1-3)αGlc CH-1
15	Sialic acid	2.06	CH_3_			5.44	Fuc (α1-3) βGlc CH-1
16	Glutamate	2.12	β-CH_2_	43	Urea	5.76	NH_2_
		2.33	γ-CH_2_	44	Fumarate	6.51	CH = CH
17	Methionine	2.13	CH_3_	45	Tyrosine	6.9	CH-3,5
18	Glutamine	2.11	β-CH_2_			7.2	CH-2,6
		2.46	γ-CH_2_	46	Methylhistidine	7.06	CH-4
19	Citrate	2.51	α-CH_2_			7.8	CH-2
		2.69	α’-CH_2_	47	Phenylalanine	7.32	CH-2,6
20	Dimethylamine	2.72	2 CH_3_			7.37	CH-4
21	2-oxogluturate	2.99	CH_2_			7.42	CH-3,5
22	Creatinine	3.02	CH_3_	48	Hippurate	7.54	CH-2,6
23	Creatine	3.03	CH_3_			7.63	CH-4
24	Malonate	3.12	CH_2_			7.82	CH-3,5
25	Choline	3.19	3 CH_3_	49	Formate	8.44	CH
26	O-Phosphocholine	3.21	3 CH_3_	50	Cytidine triphosphate	5.99	CH-2
		4.16	CH_2_			6.13	CH-10
27	Carnitine	2.42	CH_2_			7.97	CH-11
		3.21	3 CH_3_	51	Uridine	5.89	CH-2
28	Betaine	3.24	3 CH_3_			5.91	CH-10
						7.87	CH-11

Abbreviations: 3-BHBA, 3-betahydroxybutyrate; FL, fucosyllactose; Fuc, Fucose; GlcNAc, *N*-acetylglucosamine; LDFT, lactodifucotetraose; LNDFH, lacto-*N*-difucohexaose, LNFP, lacto-*N*-fucopentaose; SL, sialyllactose.

## Abbreviations

The following abbreviations are used in this manuscript:

HM: Human milk
HMO: Human milk oligosaccharide
NMR: Nuclear magnetic resonance
